# JMJD family proteins in cancer and inflammation

**DOI:** 10.1038/s41392-022-01145-1

**Published:** 2022-09-01

**Authors:** Wang Manni, Xue Jianxin, Hong Weiqi, Chen Siyuan, Shi Huashan

**Affiliations:** 1 Department of Biotherapy, Cancer Center, West China Hospital, Sichuan University, No. 17, Block 3, Southern Renmin Road, 610041 Chengdu, Sichuan PR China; 2 Department of Thoracic Oncology, Cancer Center and State Key Laboratory of Biotherapy, West China Hospital, Sichuan University, Chengdu, China; 3 Laboratory of Aging Research and Nanotoxicology, State Key Laboratory of Biotherapy, National Clinical Research Center for Geriatrics, West China Hospital, Sichuan University, 610041 Chengdu, Sichuan PR China

**Keywords:** Cancer, Tumour biomarkers

## Abstract

The occurrence of cancer entails a series of genetic mutations that favor uncontrollable tumor growth. It is believed that various factors collectively contribute to cancer, and there is no one single explanation for tumorigenesis. Epigenetic changes such as the dysregulation of enzymes modifying DNA or histones are actively involved in oncogenesis and inflammatory response. The methylation of lysine residues on histone proteins represents a class of post-translational modifications. The human Jumonji C domain-containing (JMJD) protein family consists of more than 30 members. The JMJD proteins have long been identified with histone lysine demethylases (KDM) and histone arginine demethylases activities and thus could function as epigenetic modulators in physiological processes and diseases. Importantly, growing evidence has demonstrated the aberrant expression of JMJD proteins in cancer and inflammatory diseases, which might serve as an underlying mechanism for the initiation and progression of such diseases. Here, we discuss the role of key JMJD proteins in cancer and inflammation, including the intensively studied histone lysine demethylases, as well as the understudied group of JMJD members. In particular, we focused on epigenetic changes induced by each JMJD member and summarized recent research progress evaluating their therapeutic potential for the treatment of cancer and inflammatory diseases.

## Introduction

As one of the major causes of mortality worldwide, cancer challenges global public health. According to cancer statistics 2018, one in every five men and one in every six women would develop cancer during their lifetime.^1^ Moreover, there were approximately 19.3 million new cancer cases and 10.0 million cancer deaths in 2020.^2^ The occurrence of cancer entails a series of genetic mutations that favor uncontrollable tumor growth. It is believed that various factors collectively contribute to cancer, and there is no one single explanation for tumorigenesis. For instance, cancers can be caused by internal factors such as spontaneous DNA mutations or external environmental factors. Epigenetic alterations are a class of features common to cancer progression, reversibly modulating oncogenesis through chromatin compaction, and are susceptible to external or internal environmental factors.^3^ The term “epigenetics” was originally introduced by Dr. Waddington to describe the hereditary alterations in cell phenotypes that were independent of DNA sequence change.^4^ Following decades of research, the definition of epigenetics has reached a consensus that epigenetics is the chromatin-based event that modulates DNA-templated processes.^5^

Composed of DNA and the surrounding nucleosomes, chromatin is a constantly-changing structure that responds to external environments. Each nucleosome contains an octamer of four histones (H2A, H2B, H3, and H4), the post-translational modifications (PTMs), which influences chromatin compaction and subsequently modulates the transcription levels of different genes.^6^ The histone modifications at specific residues include acetylation, methylation, phosphorylation, citrullination, ubiquitination, ADP-ribosylation, deamidation, formylation, O-GlcNAcylation, propionylation, butyrylation, crotonylation, and proline isomerization, controlling gene expression during the development of diseases.^7^ It is thus not surprising that molecules regulating the deposition and removal of histone modifications are actively involved in oncogenesis and inflammation response.^8,9^

**Fig. 1:**
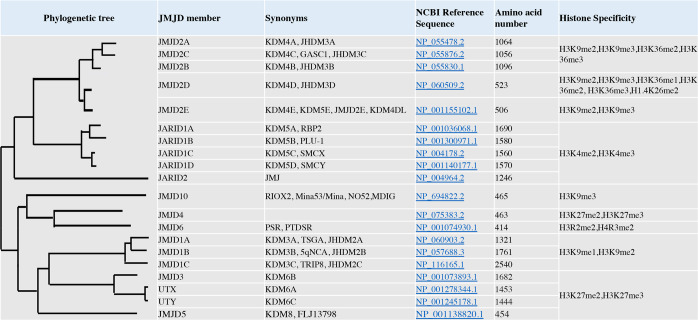
Phylogenetic tree of histone demethylase members of JMJD family proteins. Figure was created with Biorender (www.bioender.com)

The methylation of lysine residues on histone proteins represents a class of PTMs. Lysine residues of histones can be either mono-, di-, or tri-methylated (Kme1, Kme2, and Kme3, respectively) by enzymes that recognize methyl marks on histone proteins. The different methylation status of histones leads to the recruitment of binding proteins with varying affinities.^10,11^ The methylation process of lysines is accomplished by the histone lysine methyltransferases (KMTs), also referred to as “epigenetic writers”, whereas the removal of methyl groups on lysine relies on lysine demethylases (KDMs), referred to as “erasers”.^12,13^ KDMs are classified into two families according to their action mechanism, the flavin adenine dinucleotide (FAD)-dependent amine oxidases and the Jumonji C (JmjC) domain-containing (JMJD) demethylases. Figure 1 presents the phylogenetic tree of histone demethylase members of JMJD family proteins.

The human JMJD protein family consists of more than 30 members, most of which have been identified with histone lysine demethylase activity. The JMJD family of KDM enzymes function as Fe2+ and 2-oxoglutarate-dependent dioxygenases and are able to demethylate histone lysines at different methylated states (Kme1, Kme2, and Kme3).^14,15^ The signature structure of JMJD proteins is a ~170 amino acids long Jumonji C (JmjC) domain where their complexation with Fe2+ occurs.^15,16^ The activity of JMJD proteins requires the involvement of cofactors oxygen and 2-oxoglutarate (or α-ketoglutarate), resulting in the sensitivity of JMJD activity to the metabolic changes within cells.^17^ Two consecutive chemical reactions are implicated in the lysine demethylation process, including the hydroxylation of the methylated ε-amino group and formaldehyde release. In addition to methylated lysine residues, JMJD proteins also demonstrate their hydroxylation activities on amino acid residues of aspartate, asparagine, histidine, arginine, and unmethylated lysine, and tRNA.^18^

**Fig. 2:**
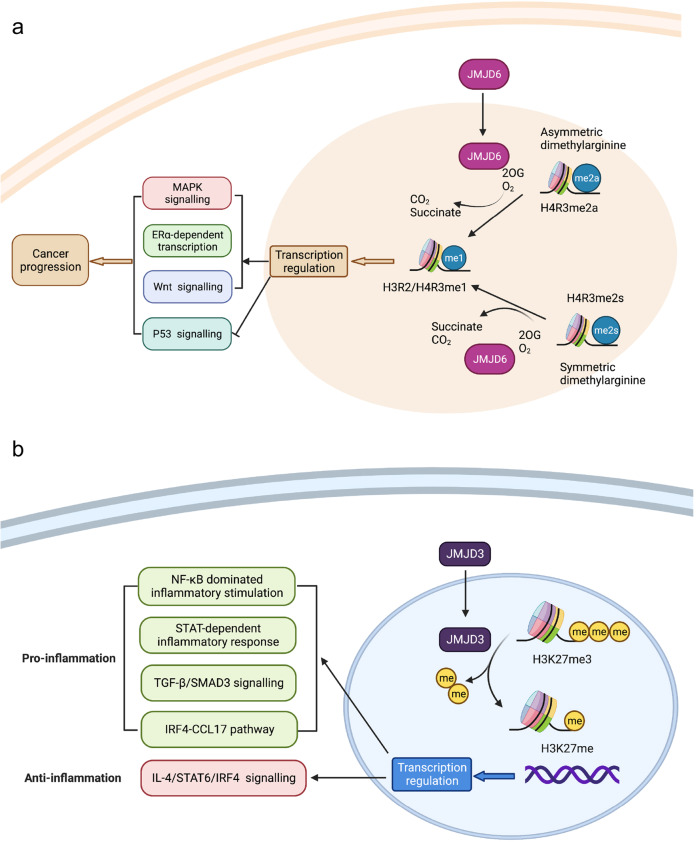
Representative diagram of the demethylating activities of JMJD family members in cancer and inflammation: JMJD3 demethylates tri-methylated Lys 27 on histone H3 and thus affects the transcription of inflammation-associated gene. JMJD6 is a 2OG oxygenase and catalyze methylarginine demethylation of histone H3/H4 residues, which regulates the transcription of cancer-related pathway genes such as MAPK signaling. Figure was created with Biorender (www.bioender.com)

The first protein identified with JmjC domain-based catalytic activity was HIF1AN (hypoxia-inducible factor 1 subunit alpha inhibitor), a hydroxylase of asparagine residues.^19,20^ This has led to speculation that JMJD proteins could hydroxylate methylated lysine residues and thereby exhibit their demethylation ability.^21^ Soon thereafter, a number of JMJD proteins were detected for their histone lysine demethylase activities, which collectively form a large heterogenous JMJD protein family.^14,22^ The classification of JMJD family members can be based on their molecular weight (>100 kDa or <100 kDa), the specificity of lysine demethylation, or the existence of functional domains. Considerable attention has converged on those JMJD proteins reported with histone lysine demethylase activity.^23^ Though some other JMJD family members are not catalytically active, such as JARID2, which contains amino acid mutations critical for cofactor binding, they are still essential for the multiple biological processes.^24^ In this review, we will focus on the role of JMJD family proteins in cancer and inflammation, including the intensively studied histone lysine demethylases and the understudied group of JMJD members. The representative diagram of the demethylating activities of JMJD family members in cancer and inflammation is presented in Fig. 2.

## JMJD proteins in cancer

**Fig. 3:**
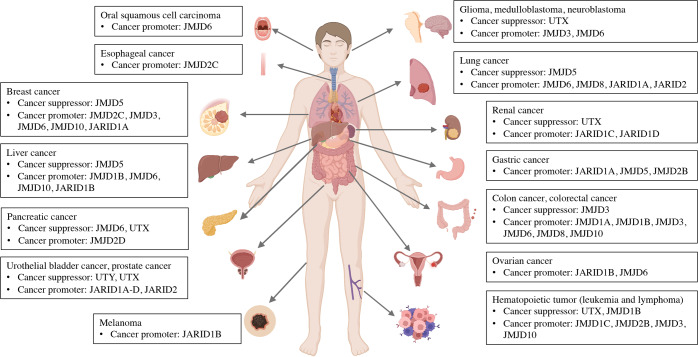
The inhibiting and promoting effects of JMJD family proteins in different cancer types. Figure was created with Biorender (www.bioender.com)

Growing evidence has demonstrated the aberrant expression of JMJD proteins in cancer and inflammatory diseases, which might serve as an underlying mechanism for the initiation and progression of such diseases. The inhibiting and promoting effects of JMJD family proteins in different cancer types are summarized in Table 1 and Fig. 3.

**Table 1:** The inhibiting and promoting activities of JMJD family proteins in cancer

JMJD protein	Cancer promoter	Cancer suppressor	Mediated signaling [Reference]	Mediated genes [Reference]
**JMJD1A**	Colorectal, prostate, hepatic and renal cancer		Wnt/β-catenin signaling,^32,34,35^ AR-V7 formation^41^	HOXA1,^37^ c-Myc,^39^ RUNX3^31^
**JMJD1B**		Acute myeloid leukemia		PML/RARα degradation^43^
**JMJD1C**	Acute myeloid leukemia		ATF2 signaling^48^	YAP1^49^
**JMJD2A**	Breast, colon and lung cancer		Akt-mTOR signaling^70^	CHD5,^67^ SLUG,^69^ PDK1^70^
**JMJD2B**	Hodgkin lymphoma, gastric and breast cancer		β-catenin signaling,^86^ TRAF6-mediated AKT activation,^83^ HOXC4/PD-L1 axis^87^	LC3B^85^
**JMJD2C**	Esophageal and breast cancer, medulloblastoma			Downstream target of Oct4^54,55^
**JMJD2D**	Pancreatic		HIF1 signaling^95^	β-catenin target genes,^94^ mTOR,^95^ PD-L1^96^
**JMJD3**	Hodgkin lymphoma, diffuse large B-cell lymphoma, breast, colon rectal, lung, prostate cancer, glioma	Colon rectal cancer	TGF-β-Smad signaling,^112^ Wnt/β-catenin signaling,^115^ STAT3 signaling,^126^ B-cell receptor signaling^141^	ZEB1,^117^ ZEB2,^117^ SNAI1,^117^ CXCL9,^121^ CXCL10,^121^ O-methylguanine-DNA methyltransferase (MGMT),^137^ transformer 2 alpha homolog (TRA2A),^137^ 2 small nuclear RNA auxiliary factor 1 (U2AF1),^137^ and ribosomal protein S6 kinase A2 (RPS6KA2),^137^ HOX,^143^ v-myc myelocytomatosis viral related oncogene (MyCN)^144^
**JMJD4**	Renal cancer			Eukaryotic release factor 1 (eRF1)^18^
**JMJD5**	Gastric adenocarcinoma	Breast, lung cancer, hepatocellular carcinoma	p53/NF-κB signaling^164^	PKM2-HIF−1α target genes,^165^ c-Myc,^167^ cancer upregulated gene 2 (CUG2)^166^
**JMJD6**	Breast, colon, lung and ovarian cancer, hepatocellular carcinoma, neuroblastoma, oral squamous cell carcinoma	Pancreatic cancer	AR-V7 formation^191^	Estrogen receptor α (ERα), tumor necrosis factor receptor‐associated factor 6 (TRAF6), and the transcription factor PAX3 and heat‐shock protein 70 (HSP70),^179–182^ E2F2, N-Myc and c-Myc,^188,189^ P53^200^
**JMJD7**	Head and neck squamous cell carcinoma			Mixed-lineage leukemia gene (MLL)^170^
**JMJD8**	Colon rectal, lung cancer	Head and neck squamous cell carcinoma	AKT/NF-κB/COX-2 signaling,^208^ NF-κB signaling,^210^ PI3K/AKT signaling^211^	
**JMJD10**	Breast, colon cancer, lymphoma, hepatocellular carcinoma		JMJD10/H3K9me3/p21 signaling^233^	
**JARID1A**	Lung, gastric, breast and prostate cancer		p16/p27-mediated senescence^244^	Integrin β-1 (ITGB1),^240^ p16, p21, and p27^249^
**JARID1B**	Melanoma, prostate, hepatocellular, head and neck, and ovarian cancer			
**JARID1C**	Clear cell renal cell carcinoma, prostate cancer	Human papilloma virus-related malignancies		Suppress E6 and E7 viral oncoprotein^271^
**JARID1D**	Clear cell renal cell carcinoma, prostate cancer		Androgen receptor signaling^274^	
**JARID2**	Lung and bladder cancer	Hematopoietic tumor	TGF-β signaling,^292^ LINC021/IMP2 signaling^293^	
**UTX**		Highly mutated in acute lymphoblastic leukemia, chronic myelomonocytic leukemia, medulloblastoma, pancreatic, bladder, prostate and renal cancer,		EZH2,^312^ KIF 14 and pAKT,^313^ SNAI and ZEB1/2^315^
**UTY**		Urothelial bladder cancer

### JMJD1

The JMJD1 subfamily includes JMJD1A, JMJD1B, and JMJD1C (or KDM3A, B, and C, respectively), which all contain 2 conserved domains required for catalytic activities,^25,26^ JmjC catalytic center, and a C6 zinc finger. JMJD1A is 59.64% identical to JMJD1B in terms of amino acids. However, both JMJD1A and JMJD1B share less than 50% of their amino acid sequences with JMJD1C, suggesting that JMJD1C is evolutionarily distinct.^18^ Though these JMJD1 proteins have been reported to demethylate mono- and dimethylated lysine 9 on histone H3 (H3K9), the trimethylated H3K9 is not a substrate for JMJD1 proteins.^27^ It was recently suggested that the demethylation activity of JMJD1 might include more histone lysines in addition to H3K9.^28,29^ JMJD1 proteins have long been found to regulate normal homeostasis, and here we mainly focus on their roles in oncogenesis.

On one hand, JMJD1A functions as a tumor-suppressive factor, with Jmjd1a knockout resulting in increased microvessel formation and the expression alterations of angiogenesis-related genes, such as the downregulation of anti-angiogenic factors.^30^ JMJD1A also suppresses the proliferation of gastric cancer cells by regulating its target gene runt-related transcription factor 3 (RUNX3).^31^ On the other hand, the overexpression of JMJD1A was found to correlate with increased metastasis and unfavorable prognosis of colorectal cancer (CRC) and gastric cancer.^32,33^ JMJD1A may promote CRC progression via Wnt/β-catenin signaling where it coactivates downstream targets of β-catenin^32,34,35^ or coactivating STAT3 transcription factor.^36^ JMJD1A expression is significantly elevated in bladder carcinomas compared with adjacent noncancerous tissues, which promotes the G1/S cycle transition by regulating HOXA1 gene transcription.^37^ Another mechanism through which JMJD1A promotes urinary bladder cancer progression is the increased glycolysis induced by JMJD1 through coactivation of HIF1α.^38^

In prostate cancer, JMJD1A promotes the proliferation and survival of prostate cancer cells via the regulation of c-Myc expression at both transcriptional and post-translational levels.^39^ In other words, JMJD1A not only enhances c-Myc transcriptional activity but also prevents the degradation of c-Myc protein.^39^ JMJD1A was found to promote the expression of factors that mediate DNA repair and radioresistance of prostate cancer cells, making JMJD1A a potential therapeutic target to improve the response of prostate cancer cells to chemotherapies, radiotherapies, and PARP inhibitors.^40^ JMJD1A promotes the formation of alternative splicing of AR variant 7 (AR-V7), a key mechanism by which prostate cancer cells develop resistance to androgen deprivation therapy.^41^

JMJD1B is located in the 5q31 chromosomal locus, the deletion of which is often seen in myelodysplasia and acute myeloid leukemia (AML). Lower expression of JMJD1B in AML patients indicated worse prognosis,^42,43^ and JMJD1B was thus viewed as a tumor suppressor for AML.^44^ The underlying mechanism may be the JMJD1B-facilitated degradation of PML/RARα, a critical event in the pathogenesis of acute promyelocytic leukemia (APL).^43^ JMJD1B also appears as a tumor suppressor in CRC, the histone methylation of which is regulated by PRL-3, an essential metastasis gene of CRC.^45^

JMJD1C was initially identified in undifferentiated spermatogonia, the knockout of which resulted in increased apoptosis of germ cells in mice.^46^ In the field of cancer, JMJD1C functions as an oncogenic factor for AML by promoting cell survival and self-renewal. For example, intracranial germ cell cancer was characterized by germline missense mutations in JMJD1C.^47^ JMJD1C is overexpressed in colon cancer tissues and increases colon cancer metastasis via the inactivation of the ATF2 pathway.^48^ On the contrary, JMJD1C functions as a tumor suppressor in esophageal cancer, which downregulates cancer cell proliferation by targeting YAP1 gene expression via H3K9me2 demethylation.^49^ Interestingly, in JMJD1C-knockout mice, the global H3K9 methylation level remained unchanged, and it thus is postulated whether JMJD1C displays H3K9 demethylase activity.^50^

### JMJD2/KDM4

JMJD2 is one of the largest JMJD subfamilies and is comprised of JMJD2A-D proteins, also referred to as KDM4A-D. JMJD2 family members take active parts in multiple physiological processes, including cell proliferation, migration,^51^ gene transcription,^52^ and genome stability.^53^ In embryonic stem cells (ESCs), KDM4B and KDM4C interact with pluripotency factors such as Sox2, Oct4, c-Myc, and Klf4, thereby regulating cell proliferation and stem-cell features.^54,55^ It was recently reported that JMJD2A, B, and C were all crucial to the survival of acute myeloid leukemia cells,^56^ but the individual role of each member varied significantly.

JMJD2A uses trimethylated H3K9 and H3K36 as demethylating substrates. Interestingly, demethylating efficiency of JMJD2A on H3K9me3 is 5-fold higher than on H3K36me3 and higher on trimethylated than dimethylated H3K9/H3K36.^57^ The dual role of JMJD2A in transcription, with both stimulating and repressing effects on gene transcription, has drawn considerable research attention. JMJD2A may directly bind to transcription factors^58^ or interact with nuclear receptor corepressor to suppress gene transcription.^59,60^ The oncogenic effect of JMJD2A was first observed in breast cancer, with approximately 60% of breast tumors identified with JMJD2A overexpression.^61–63^ JMJD2A is also important for androgen and estrogen receptor (ER) activities based on its catalytic activity.^64^ The absence of JMJD2A in ER-positive or ER-negative breast cancer cells decreased the expression of ER target genes such as the c-Jun and cyclin D1, leading to aberrant cell proliferation.^65^ A recent meta-analysis revealed the differential expression of JMJD2 in breast cancer, with JMJD2A/D overexpression predominantly observed in basal-like breast cancer and JMD2B in ER-positive luminal-type breast cancer.^66^

JMJD2A also plays a functional role in a number of other cancers. In lung cancer, JMJD2A decreased the transcription of tumor suppressor gene CHD5 to block cellular senescence, which ultimately stimulated cellular transformation.^67^ Likewise, the upregulation of JMJD2A expression was later reported in prostate cancer and bladder cancer tissues.^68^ In bladder cancer, JMJD2A promoted epithelial-mesenchymal transition (EMT) by modulating SLUG expression. However, contradictory results were reported that lower JMJD2A intensity was observed in bladder cancer tissue samples, predicting significantly worse overall survival.^69^ JMJD2A promoted the growth and protein synthesis of gliomas via phosphoinositide-dependent kinase-1 (PDK1)-mediated Akt-mTOR pathway activation.^70^ Notably, there appeared to be no difference between JMJD2A upregulation or downregulation in the growth of cervical carcinoma.^71^ These results suggested that JMJD2A might preferentially stimulate the growth of specific tumor types.

JMJD2B and JMJD2C share similar structures and action specificity to JMJD2A.^72^ It remains unclear whether the catalytic activity of JMJD4B is lower than other JMJD2 members because the different sizes of recombinant JMJD4B proteins would affect the measurement results.^73^ It is widely accepted that JMJD2B supports the carcinogenesis of ER-positive tumors because it is, in fact, an ER target gene.^74–76^ Though JMJD2B/C is upregulated in breast cancers at mRNA levels, higher JMJD2B expression was observed in ER-positive than ER-negative breast cancer, whereas the reverse applied for JMJD2C.^77,78^ As a downstream target of the pluripotency factor Oct4, JMJD2C promoted not only the proliferation of ER-negative breast cancer cells but also cancer-stem-cell features such as mammosphere formation in non-transformed breast cancer cell lines,^79^ suggesting the important role of JMJD2C in the maintenance of cancer stem cells. JMJD2B is associated with invasion and metastasis of gastric cancer by inducing EMT.^80^ JMJD2B and β-catenin collectively promote the transcription of β-catenin target gene vimentin via H3K9 demethylation.^80^ The overexpression of JMJD2B was found to correlate with the abundance of p-c-Jun in gastric cancer, which is predictive of poor survival.^81^ In classical Hodgkin lymphoma, the elevated expression of JMJD2B and JMJD2D was also associated with aggressive subtypes and suboptimal treatment response to radiation.^82^

In CRC patients, the overexpression of JMJD2B in CRC specimens indicates a poor prognosis. It has been demonstrated that JMJD2B accelerated CRC progression based on its interaction with TRAF6, which leads to TRAF6-mediated AKT activation.^83^ A more recent study identified a novel epigenetic mechanism for the progression of CRC, where JMJD2B enhances the transcription of small GTPase TC10-like (TCL), leading to a malignant phenotype of CRC cells.^84^ Additionally, under glucose deficient conditions, JMJD2B sustained the autophagy-derived amino acids in CRC cells via the epigenetic regulation of LC3B, thereby promoting the aggressiveness of CRC.^85^ Similar to its action mechanism in gastric cancer, JMJD2B supports the gene transcription induced by β-catenin, thereby contributing to the tumorigenesis of CRCs.^86^ The invasion of CRC cells may also be attributed to the immune escape via the KDM4B/HOXC4/PD-L1 axis.^87^

A unique member of the JMJD2 family, JMJD2D, is only half the size of JMJD2A-C due to its lack of PHD and Tudor domains.^88^ Compared with JMJD2A-C, which uses H3K36 as demethylating substrates, JMJD2D has a different substrate-binding specificity and acts on dimethylated H1.4K26 rather than trimethylated H1.4K26.^89^ JMJD2D also demethylates H3K9me2 and H3K9me3 but less efficiently demethylates H3K9me1.^90,91^ The expression of JMJD2D in the margins of pancreatic tumors was indicative of earlier recurrence in patients.^92^ JMJD2D is highly expressed in liver cancer, and its demethylase-independent inhibition on p53 tumor suppressor promotes liver cancer initiation and progression.^93^ The chemical inhibition of JMJD2 by ML324 enhances cell apoptosis of hepatocellular carcinoma via the unfolded protein response and Bim upregulation.^93^

Compared with noncancerous colon tissues, JMJD2D is highly expressed in CRC tissues and promotes tumor growth and invasion of CRC. The crosstalk between JMJD2D and β-catenin activates the transcription of β-catenin target genes in CRC cells.^94^ The colon tumorigenesis by JMJD2D can be mediated by Hedgehog signaling. In addition, a recent study suggested that JMJD2D enhanced CRC progression by activating HIF1 signaling and subsequent cell glycolysis.^95^ The activation of the HIF1 signaling pathway by JMJD2D can be based on three mechanisms: (1) JMJD2D upregulates mTOR expression, thus promoting HIF1α translation; (2) JMJD2D upregulates HIF1β transcription; (3) JMJD2D interacts with HIF1α to induce glycolytic gene expression.^95^ JMJD2D is also involved in the immune escape of CRC cells by upregulating PD-L1 expression, providing a new strategy to improve response to anti-PD-1/PD-L1 immunotherapies.^96^ Bioinformatical analyses revealed that for two potential JMJD2 members, the gene products of JMJD2E and JMJD2F were similar to those of JMJD2D, but JMJD2E and JMJD2F are more likely to be pseudogenes.^97^

Importantly, the role of JMJD2/KDM4 proteins in tumorigenesis is especially addressed in colorectal cancer. JMJD2A promoted cell growth of colon cancer by increasing cell proliferation and at the same inhibiting apoptosis.^58^ JMJD2B is also overexpressed in CRC tissues, the inhibition of which promotes cell apoptosis providing a potential therapeutic strategy.^98^ JMJD2B could be induced under a hypoxic environment in a HIF-1α-dependent manner in CRC cells. Under such circumstances, the expression of several hypoxia-inducible genes was upregulated by JMJD2B via demethylation of H3K9me on their promoters.^99^ It was recently suggested that Wnt-mediated CRC metastasis is partially dependent on JMJD2 to form an epigenetic complex that activates disintegrin and metalloproteinase (ADAM) transcription.^100^

### JMJD3/KDM6B

The JMJD3 gene is located on chromosome 17p13.1^101,102^ and 88% homologous to the gene histone demethylase gene UTX (ubiquitously repeat transcribed tetratricopeptide repeat on the X chromosome).^103^ UTX was the first identified mutated histone demethylase gene associated with cancer^104^ and can remove the methyl groups from di-trimethylated H3K27.^103,105–107^ The location of JMJD3 is adjacent to p53 as well, a tumor suppressor, the mutation of which is a frequent event in cancer. It was found that JMJD3 might also directly interact with p53.^108,109^ The role of JMJD3 on cancer is highly controversial, with tumor inhibitory effects on CRC, hepatic cancer, pancreatic cancer, glioma and B-cell lymphoma, and tumor-promoting effect on cancers such as renal breast, prostate, and ovarian cancer.

The regulating effect of JMJD3 in cancer is partially attributed to its role in the EMT of cancer cells. Transforming growth factor-β (TGF-β) is a well-characterized multipotent cytokine that inhibits tumor cell proliferation at the early stage but induces EMT at the late stage of cancer progression.^110^ The increased expression of JMJD3 was proved to be associated with metastatic capacities of ovarian cancer via the increased expression of TGF-β.^111^ Moreover, JMJD3 promoted EMT of Ras-mutated lung cancer cells via TGF-β-mediated Smad stimulation.^112^ In line with the in vitro studies, non-small cell lung cancer (NSCLC) patients with high JMJD3 expression in tumor specimens displayed higher risks of lymphatic and distant metastasis and poor overall survival.^113^ Further studies provided a potential mechanism through which JMJD3-mediated EMT of cancer cells. JMJD3 enhanced TGF-β-induced EMT by upregulating the EMT-related gene, SNAI1, in invasive breast cancer.^114^

Previous evidence also investigated the important yet poorly defined role of JMJD3 in the metastasis of CRC, the second most lethal cancer worldwide in 2020.^2^ The aberrant expression of Wnt/β-catenin pathway molecules is often observed in a wide spectrum of cancers and is believed to be associated with epigenetic modulation of key gene promoters in colon cancer.^115^ JMJD3 has a dual role in CRC as a tumor suppressor and tumor activator. JMJD3 is under-expressed in CRC patient specimens, and the absence of the JMJD3 in the tumor is characterized as a marker of poor clinical outcome in CRC.^116^ Earlier evidence identified JMJD3 as a downstream target of vitamin D metabolite 1α,25-dihydroxyvitamin D(3) (1,25(OH)(2)D(3)) in colon cancer cells which mediates the effects of 1,25(OH) 2D 3 on a subset of EMT-inducer genes such as ZEB1, ZE, B2, and SNAI1.^117^ The expression of JMJD3 was found to inversely correlate with that of SNAI1 in colon cancer tissues, and the inhibition of JMJD3 abolished 1,25(OH)(2)D(3)-induced β-catenin transcriptional activity.^118^ On the other hand, JMJD3 promotes the expression of the epithelial cell adhesion molecule (EpCAM) gene in CRC based on its histone promotor demethylation function.^119^ The aberrant activation of NOTCH1, and the subsequent increase in Ephrin type-B receptor 4 (EPHB4) expression, are considered a hallmark of CRC progression. The intracellular NOTCH domain led JMJD3 to the EPHB4 enhancer region, and modified the chromatin architecture by regulating the H3K27me3 level, which ultimately resulted in EPHB4 activation.^120^ Recently, JMJD3 has been reported to control tumor immunosuppression. JMJD3-mediated H3K27me3 reduced the production of Th1-type chemokines CXCL9 and CXCL10, mediators of effector T-cell trafficking in colon cancer.^121^

In accordance with the tumor-supportive action of JMJD3 in CRC, the regulation of JMJD3 on cancer development based on its demethylation activities can also be found in a series of other cancer types. The brain is the most investigated organ for JMJD3 regulation. Patients with pediatric brainstem glioma often experience a decrease in H3K27me3, and the methylation maintenance by JMJD3 inhibition thus becomes an important treatment strategy.^122,123^ JMJD3 expression was elevated in glioma relative to normal tissues, and the inhibiting JMJD3 demethylation reduced tumor cell proliferation and migration, and at the same time enhanced apoptosis.^124^ In contrast, some reports addressed JMJD3 as a tumor suppressor in glioma via modulating the expression of the key transcription factors, including the p53.^125^ As discussed earlier, JMJD3 may directly interact with p53 and regulate its activity independently of chromatin modification, leading to glioblastoma stem-cell (GSC) differentiation.^108^ Moreover, JMJD3 expression is partially determined by STAT3 (signal transducer and activator of transcription 3), which binds to and inhibits the promotor region of JMJD3. Once JMJD3 expression was resumed from STAT3 inhibition, JMJD3 reduced the formation of neurosphere and cell proliferation of GSC.^126^ One of the treatment strategies for glioblastoma is an aptamer which targets the ligand of platelet-derived growth factor receptor A (PDGFRα), leading to decreased STAT3 and increased JMJD3 expression, and subsequent upregulation of p53.^127^ Collectively, these results suggested the central position of JMJD3 in the STAT3-JMJD3-p53 signal network that regulates glioma progression.^128^

The occurrence of prostate cancer (PC) is highly relevant to histone modifications such as methylation,^129^ and the fact that an H3K27 methyltransferase has been found to indicate prostate cancer progression further supports the oncogenic role of histone methylation status at H3K27 in prostate cancer.^130^ PC is hormone-dependent cancer with overexpression of androgen receptor (AR).^131^ A wide breadth of literature has suggested the link between JMJD3, H3K27me3, and the AR metabolic pathway.^132–136^ Increased transcriptional level of JMJD3 was reported in metastatic prostate cancer,^105^ with higher expression in AR-positive compared to AR-negative PC cell lines.^132^ The signature genes activated by JMJD3 in PC include (O-methylguanine-DNA methyltransferase (MGMT), transformer 2 alpha homolog (TRA2A), and 2 small nuclear RNA auxiliary factor 1 (U2AF1), and ribosomal protein S6 kinase A2 (RPS6KA2)), identified as signature genes in PC.^137^

The overexpression of JMJD3 is often observed in germinal center B (GC-B) cells in Hodgkin lymphoma (HL)^138^ and diffuse large B-cell lymphoma (DLBCL).^139,140^ Following JMJD3 blockade treatment, the H3K27me3 level on target genes was significantly decreased in HL, supporting the conclusion that JMJD3 is involved in the development of HL.^138^ In DLBCL, JMJD3 promotes the phosphorylation of proteins mediating the B-cell receptor (BCR) signaling. It affects its downstream B-cell lymphoma 6 protein (BCL6), facilitating normal B-cell survival and lymphogenesis of B-cell non-Hodgkin lymphoma (NHL).^141^ Moreover, JMJD3 is involved in the treatment response to chemotherapies, with JMJD3 inhibitors demonstrating significant chemo-sensitization on B cells.^139^ Recently, a potential link was established between JMJD3 demethylase and cyclin-dependent kinase 9 (CDK9), the abnormal expression of which was a frequent event in DLBCL. The use of CDK9 inhibitors reduced JMJD3 expression, which specifically elevated the trimethylation of H3K27.^142^

JMJD3 is also involved in a broad spectrum of cancers such as PML/RARα-positive leukemic, where JMJD3 activates expression of the homeobox (HOX) gene via interaction with PML-RARα fusion protein.^143^ In neuroblastoma which is mainly induced by the activation of oncogenes and the failure of neural crest cell differentiation, blocking JMJD3 may regulate the expression of several key differentiation genes such as the v-myc myelocytomatosis viral-related oncogene (MyCN).^144^ Based on these intriguing findings, further studies are warranted to clarify the precise role and action mechanisms of JMJD3 in cancer under different circumstances.

### JMJD4

JMJD4 is a recently identified histone demethylase homologous to JMJD6. However, compared with JMJD6, which has been intensively studied, experimental work to characterize the role of JMJD4 in cancer has lagged far behind. Currently, the only reported enzymatic activity of JMJD4 is the hydroxylation of the lysine residue of eukaryotic release factor 1 (eRF1).^18^ Though the inhibition of eRF1hydroxylation led to less efficient transcriptional termination, researchers failed to identify any physiological consequences.^145^ An initial investigation suggested significantly higher JMJD4 expression in tumor tissues than in normal tissues of the colon and hepatic cancer and the differential expression of JMJD4 protein in colon cancer of different histological grades and metastasis status.^146^ Recent research revealed the increased expression of JMJD4 expression in renal cancer, which might be a prognostic marker in renal cancer patients.^147^ Thus, more studies are needed to delineate the role of JMJD4 in cancer.

### JMJD5 and JMJD7

JMJD5/ KDM8 shuttles between the cytoplasm and the nucleus^148^ exhibit a wide range of enzymatic activities, including the demethylation of H3K36me2,^149,150^ hydroxylation at the C3 of arginine residues,^151–153^ and proteolysis.^154,155^ Recent research has cast doubt on the demethylation of JMJD5 H3K36me2 based on the crystal structure results that the catalytic center of JMJD5 is not favorable to the accommodation of methylated lysine residues.^151,156–158^ Further, no valid in vivo evidence has been reported for the arginine hydroxylation activities of JMJD5.^153^ In keeping with JMJD5, JMJD7 is able to hydroxylate the C3 position of lysine residues and at the same time, cleaves arginine methylated histone as a protease.^154^ The hydroxylation of DRG1/2, two GTPases involved in ribosome biogenesis,^159^ by JMJD7 enhanced the binding of DRG1/2 to RNA. Noteworthy, the occupancy of JMJD7 at gene promoter regions negatively regulates osteoclast differentiation, suggesting the critical role of JMJD7 in bone formation and turnover.^160^ The divalent cation-dependent protease activities of JMJD5 and JMJD7 preferentially cleave the tails of H2, H3, and H4 bearing methylated arginine. Like other aminopeptidases, JMJD5 and JMJD7 digest the C-terminal products following the initial specific cleavage, providing a new repertoire for removing histone tails with methylated arginine residue.^154^ Interestingly, histones such as H3 and H4 and their arginine methylated isoforms were increased in cells lacking either JMJD5 or JMJD7 in vivo.^154^

JMJD5 is highly expressed in breast cancer cell lines, the knockdown of which resulted in tumor cell growth arrest.^149^ It was recently suggested that there is significantly lower mRNA expression of JMJD5 in breast cancer, hepatocellular carcinoma, and lung cancer, but a higher expression in stomach adenocarcinoma than in normal tissues. Accordingly, high JMJD5 expression indicated a good prognosis in breast cancer, hepatocellular carcinoma, and lung cancer but a poor prognosis in stomach adenocarcinoma.^161^ In this study, JMJD5 expression was also related to the abundance of infiltrating immune cells in tumors, which might jointly serve as a prognostic marker.

In cancer cells, JMJD5 promotes the transcription of PKM2-HIF-1α target genes that mediates glucose metabolism, leading to increased glucose uptake and lactate secretion.^162^ JMJD5 has been identified as a binding partner for p53 tumor suppressor and positively regulates cell proliferation and cell cycle.^163^ Thus, in oral squamous cell carcinoma, the downregulation of JMJD5 significantly induces apoptosis and reduces tumor metastasis via p53/NF-κB pathway.^164^ A recent study described potential mechanisms for the regulation of both androgen-responsive and metabolic genes by JMJD5 in castration-resistance of prostate cancer (CRPC) cells. JMJD5 can either interacts with androgen receptor (AR) and modulates androgen response, or with PKM2 to regulate tumor metabolism under androgen-deprived conditions.^165^ Moreover, JMJD5 inhibition prevented cancer-stem-cell-like mediated by cancer upregulated gene 2 (CUG2).^166^ In pancreatic cancer however, JMJD5 negatively regulates c-Myc expression, which suppresses tumor cell proliferation and glycolytic metabolismr.^167^

Likewise, deletion of JMJD7 also impaired the viability of prostate cancer cells,^168^ and reduced colony formation of breast cancer cells.^154^ JMJD7 also promoted cell survival of head and neck squamous cell carcinoma (HNSCC) by modulating the phosphorylation of protein kinase B.^169^ It was recently hypothesized that the loss of function of mixed-lineage leukemia gene (MLL) 1 fusion, a major cause of pediatric leukemia, coupled with the failed conversion of H3K4me1 to H3K4me3, might trigger the malignant transformation of cells, suggesting a potential role of JMJD5 and JMJD7 in leukemia development.^170^

### JMJD6

JMJD6 is a 47.5 kDa protein with 403 amino acids. JMJD6 is a monomer and can be in the trimeric, pentameric or larger oligomeric form in solution and in fibril form in the absence of its poly-Ser sequence.^171^ Earlier reports refereed JMJD6 as a surface marker on macrophages, fibroblasts and epithelial cells, originally named PSR (phosphatidylserine receptor).^172,173^ Later studies found that JMJD6 was in fact predominantly located in the cellular nucleus both in cells endogenously expressing JMJD6 and JMJD6-transfected cells.^174^ As PSR was further proved as a nuclear 2-oxoglutarate (2OG)-and Fe(II)-dependent oxygenase,^175^ it was later renamed to JMJD6.^176^ In mouse models, JMJD6 deficiency resulted in neonatal lethality and serious defects in the development of organs, independent of its apoptotic cell removal activities.^177^

A key catalytic activity of JMJD6 is the arginine demethylation, both on mono-methylarginine and dimethylarginine residues of histones. To date JMJD6 is the only enzyme reported with a potential arginine demethylation activity in vivo.^178^ Recent evidence has presented non‐histone targets of arginine demethylation by JMJD6, including RNA helicase A, estrogen receptor α (ERα), tumor necrosis factor receptor‐associated factor 6 (TRAF6), and the transcription factor PAX3 and heat‐shock protein 70 (HSP70).^179–182^ However, some studies cast doubt on the function of JMJD6 as a histone arginine demethylase in cells such as endothelial cells.^183^ Researchers failed to identify arginine methylation at H4R3 in JMJD6-knockdown endothelial cells,^184^ which was further supported by the crystal structure analysis that JMJD6 structure was not conduction to demethylation activities.^185^

As a multi-functional enzyme intensively involved in chromosomal rearrangement and gene transcription, JMJD6 functions as arginine demethylase and lysyl hydroxylase,^186^ and even tyrosine kinase of histones.^187^ In glioblastoma and neuroblastoma, JMJD6 forms protein complexes with N-Myc and BRD4 (Bromodomain-containing protein 4), which is important for gene transcription of a number of genes including E2F2, N-Myc and c-Myc.^188,189^ As a tumorigenesis factor for neuroblastoma, JMJD6 is highly expressed in human neuroblastoma tissues and the knockdown of JMJD6 decreased neuroblastoma cell proliferation and tumor progression in vivo, suggesting the potential of JMJD6 as a therapeutic target in neuroblastoma.^190^ Moreover, JMJD6 is a critical regulator of AR splice variant 7 (AR-V7) which mediates the endocrine resistance in advanced prostate cancer.^191^

Accumulating evidence suggested that increased JMJD6 expression in breast cancer cells was associated with increased tumor growth and metastasis.^192–194^ According to analyses from patient tumor samples, the expression level of JMJD6 varies among breast cancer subtypes. For instance, ER‐positive tumors exhibited significantly lower JMJD6 expression than ER‐negative tumors, which explained the fact that JMJD6 was consistently related to ER‐negative diseases.^193^ However, in this study, no significant correlation between the JMJD6 level and the prognosis was identified.^193,194^ Furthermore, Claudin‐low breast tumors displayed the highest JMJD6 expression, followed by basal-like, HER2‐enriched and luminal B subtypes, with the lowest expression detected in luminal A subtype.^193^

The oncogenic role of JMJD6 in oral squamous cell carcinoma is potentially attributed to stem‐like properties mediated by JMJD6,^195–197^ which is assumed as a key factor for cancer recurrence and treatment failure.^198^ JMJD6 is crucial to melanoma progression as well, the mutation, amplification, or deletion of which indicates unfavorable prognosis.^199^ Interestingly, a study identified a novel post-translational modification of P53 by JMJD6 independent of its histone arginine demethylation activity, where JMJD6 antagonized p53 acetylation and repressed its following transcriptional activity in colon cancer.^200^ Other cancer types that have been reported to be affected by JMJD6 expression levels include lung cancer,^201,202^ hepatic cancer,^203^ and ovarian cancer,^204^ where high level of JMJD6 expression correlates with increased cell proliferation, invasiveness, and poor clinical outcomes.

### JMJD8

JMJD8 is evolutionarily distant from the other members of the JMJD family,^18^ which contains a JmjC domain at 74–269 amino acid residues with no other recognizable protein domains.^205^ JMJD8 is mainly localized at the endoplasmic reticulum and reportedly involved in angiogenesis and cell metabolism.^206,207^ Previous research has demonstrated that JMJD8 JMJD8 functions as a positive regulator of TNF-induced NF-κB signaling.^205^ The knockdown of JMJD8 upregulated AKT/NF-κB/COX-2 pathway and enhanced Ku70/Ku80 expression in cancer cells, thereby regulating cell proliferation and their responses to cancer treatments that induced DNA damage.^208^ A recent study investigated the prognostic value of 8 glycolysis-related genes in HNSCC and identified JMJD8 as a protective gene for HNSCC.^209^ Another study verified that JMJD8 functioned as an oncogene in CRC which promoted cell proliferation and EMT through the NF-κB pathway.^210^ Likewise, JMJD8 promoted carcinogenesis of NSCLC cells by maintaining EGFR stability and the downstream PI3K/AKT signaling pathway,^211^ which accorded with a recent finding that JMJD8 could modulate tumor EMT via AKT activation.^212^ Thus, JMJD8 is a potential prognostic marker and therapeutic target for cancer patients. However, JMJD8 has not been thoroughly studied and its precise role in cancer remains to be elucidated.

### JMJD10/MDIG

Given the different identification sources and multiple biological functions, JMJD10 is also frequently referred to as RIOX2, Mina53/Mina, NO52 or MDIG (mineral dust-induced gene).^213–216^ JMJD10 was initially detected in alveolar macrophages of coal miners and its expression can be induced by environmental cancer risk factors such as silica, smoke and arsenic.^217^ The inverse correlation between MDIG expression and H3K9me3 level in lung tumors^218^ supported the role of MDIG as a histone demethylase and an epigenetic regulator in a number of cancer types.^219–221^ One structural study failed to prove the histone demethylase activity of MDIG, but rather identified its hydroxylase activity toward the ribosomal protein L27a (RPL27a).^222^ For the first time, the recent study for the first time identified MDIG as an antagonist for histone methylation repressors, suggesting the potential of MDIG as a new target for cancer therapy.^223^

Many cancers have been identified with overexpression of MDIG relative to normal tissues, such as breast cancer,^224^ colon cancer,^225,226^ lymphoma.^220,227–231^ The screening results of the expression pattern of lung cancer revealed that 90 percent of lung cancers displayed elevated MDIG expression level.^230^ An underlying mechanism for MDIG-induced invasion and metastasis of lung cancer cells may be the destabilization of β-catenin and subsequent suppression of EMT-related genes.^232^ MDIG was frequently overexpressed in hepatocellular carcinoma (HCC) which was associated with higher histological grades, potentially modulating HCC progression via MDIG/H3K9me3/p21 pathway.^233^ In some cancers, higher MDIG expression is predictive of worse prognosis.^234^ Interestingly, the prognostic value of MDIG may vary in the same cancer types. For instance, increased MDIG expression is associated with longer OS in breast cancer patients with lymph node or distal metastasis.^224^ Likewise, overexpression of MDIG is indicative of prognosis only in patients at stages I/II, but not in stages III/IV patients.^216^ These results suggest that MDIG may be an oncogene that promotes tumor growth at early stages and a tumor-suppressive gene that reduces the metastatic capacity of tumor at late stages.

### JARID1(Jumonji And AT-Rich Interaction Domain Containing 1)/ KDM5

Before identifying of their histone demethylase activities, JARID1 family members were initially reported to play important roles in stem-cell biology and congenital disease. Four JARID1 members that are upregulated in cancers have been identified so far, JARID1A, JARID1B, JARID1C and JARID1D. JARID1A was first identified by screening a library of cDNA that interacted with retinoblastoma gene product (pRb),^235^ and its crosstalk with pRb reinforced the transcription repression on cell differentiation by retinoblastoma family.^236^

JARIDA is highly expressed in pediatric acute megakaryoblastic leukemia (AMKL), and its C-terminal PHD finger forms a fusion gene with NUP98.^237,238^ This fusion impairs myeloblast differentiation and promotes self-renewal of progenitor cells, which is important to leukemogenic transformation.^239^ The expression of JARIDA is also upregulated in breast cancer,^240–244^ prostate cancer,^245^ and gastric cancer,^246–249^ potentially by enhancing tumor cell proliferation and metastasis. In triple-negative breast cancer (TNBC), the anti-tumoral effect of blocking JARIDA impaired cell cycle progression and p16/p27-mediated senescence,^244^ which accorded with previous results on gastric cancer where JARID1A repressed cyclin-dependent kinase inhibitors including p16, p21, and p27.^249^ The oncogenic effect of JARIDA on PC is partially attributed to its demethylation activities on H3K4, which decreases the expression of KLF4 and E-cadherin, and facilitates cell proliferation and metastasis.^250^ JARIDA can also induce tumor metastasis of TNBC independent of its demethylase-dependent function, by promoting integrin β−1 (ITGB1) expression.^240^ Furthermore, JARIDA is responsible for the generation of therapeutic responses in cancer.^251^ One such example is breast cancer resistance to trastuzumab and erlotinib induced by JARIDA.^252^ On the contrary, JARIDA improved the treatment response of melanoma cells to immune checkpoint blockade,^253^ suggesting that the role of JARIDA in treatment response may vary based on tumor types and drug types.

JARID1B was initially regarded as an oncogene in breast cancer,^254^ the overexpression of which was significantly associated with poor prognosis,^255^ despite later research identifying its suppressive activities on the invasion of TNBS cells.^256^ JARID1B is upregulated in a wide range of cancers including prostate,^257^ hepatocellular,^258^ head and neck cancers^259^ and ovarian cancer,^260^except for melanomas with relatively low JARID1B expression.^261^ JARID1B expression has been reported to confer stem cell-like features to cancer cells,^262^ and regulate oxidative metabolism.^263^ JARID1B may also reduce the progression by suppressing the genome-wide H3K4me3 hyper-methylation in leukemias.^264^ Recent evidence suggested that JARID1-targeted inhibitors could overcome cisplatin resistance to platinum-based chemotherapeutics in melanoma.^265^ Likewise, CPI-455, the first tool compound selectively targeting the JARID1 family, inhibited the stem cell-like properties of oral cancer.^266^

JARID1C is an X-linked gene,^267^ the aberrant function of which leads to X-linked retardation.^268^ It has been well established that JARID1C plays a dual role both as a tumor promoter and a tumor suppressor. For instance, in clear cell renal cell carcinoma (CCRCC), mutations in JARID1C gene lead to its functional loss and the preferential occurrence of CCRCC in males.^269,270^ JARID1C could impair the development of papilloma virus-related malignancies by forming a complex with viral E2 that suppressed the E6 and E7 viral oncoprotein promoters.^271^

Earlier studies referred to JARID1D as a minor histocompatibility antigen on the Y chromosome.^272^ The downregulation, mutation, or loss of JARID1D was recently shown in metastatic PC and CCRCC.^273^In hormone-sensitive PC, the interaction between JARID1D and androgen receptor inhibits the transcriptional activation of AR target genes and loss of JARID1D may result in treatment failure due to dysregulating AR signaling.^274^ These efforts may only represent the tip of the iceberg regarding the role of JARID1D in cancer progression, and more studies are warranted to address the biological contributions of JARID1 to cancer.

### JARID2 (Jumonji And AT-Rich Interaction Domain Containing 2)

JARID2 is often described as and probably the most widely studied a PRC2-associated factor.^275–279^ PRC2 is a protein complex consisting of 4 core subunits, including the enhancer of zeste homolog 1 or 2 (EZH1/2), embryonic ectoderm development (EED), suppressor of zeste 12 (SUZ12) and retinoblastoma associated protein 46/48, (RbAP46/48), also known as RBBP4/7.^280^ PRC2 is responsible for the mono-, di and tri-methylation of H3K27, with approximately 70% of H3 histones being methylated by PRC2.^281–284^

Though JARID2 is a founder member of the JMJD protein family,^285^ it lacks the essential residues required for enzymatic activity, making its JmjC domain inactive.^286,287^ Since its discovery, the role of JARID2 in mammalian development has mainly converged in the embryonic stem cell pluripotency. Accumulating evidence has reported its function in embryonic lethality, based on different genetic backgrounds of the mutant strain.^288^ Apart from the JmjC domain, JARID2 contains a JmjN domain and two other domains with DNA-binding capacity which is likely to be independent of its crosstalk with PRC2.^278^

Except for the its function in the embryological context,^289,290^ JARID2 was also dysregulated in cancer and considered as an oncogene that promotes cancer progression. Through inhibiting the overactivation of AKT induced by phosphatase and tension homolog (PTEN), JARID2 facilitated EMT and invasion of HCC cells.^291^ It is thus not surprising that the knockdown of JARID2 reduced the TGF-β-mediated EMT in colon and lung cancer cells.^292^ A recent study demonstrated the essential role of the LINC021/IMP2/JARID2 signaling axis in CRC tumorigenesis where LINC021 enhanced the mRNA stability of JARID2.^293^ In bladder cancer, JARID2 promoted the proliferation, migration, invasion and sphere-forming capacities of bladder cancer cells.^294^ Previous studies suggested that tumor cells undergoing EMT are more likely to be resistant to cisplatin.^295,296^ Researchers later found that JARID2 was involved in developing cisplatin resistance in non-small cell lung cancer via upregulation of Notch1.^297^ Nevertheless, JARID2 is not always an oncogene that facilitates tumorigenesis. JARID2 can also function as a hematopoietic tumor suppressor that limits the self-renewal of multipotent progenitor cells and prevents the transformation of nonmalignant blood disorders such as myeloproliferative neoplasms and myelodysplastic syndromes, into AML.^298^

### UTX/KDM6A and UTY

KDM6A or UTX was first identified in 2007 together with JMJD3 and ubiquitously transcribed tetratricopeptide repeat on chromosome Y (UTY) as a group of H3K27 demethylases.^299,300^ Whereas UTX is an X-linked protein with demethylating activities on H3K27me2/3, UTY is the Y-linked homolog of UTX which shares similar structures with UTX with minimal demethylase activity due to a mutation in the JmjC catalytic domain.^107,301^

UTX is one of a few cancer suppressors that escape X inactivation, leading to a predominant occurrence rate in the male population.^270^ According to the analyses of 4,742 human tumor specimens.^302^ UTX is highly mutated across various cancers, including acute lymphoblastic leukemia,^303^ chronic myelomonocytic leukemia,^304^ bladder cancer,^305^ medulloblastoma,^306^ prostate cancer,^307^ and renal carcinoma.^308^ The proliferation of cancer cells was reduced when inactivating UTX mutations were resumed with the addition of wild-type UTX. An increased mutational rate of UTX was observed from 10.7% to 21.6% in pancreatic cancer samples. The knockdown or inactive mutations of UTX increased TP63 expression, which was considered a key driver of pancreatic cancer.^309^

On the contrary, specimen analysis of patients with oral tongue squamous cell carcinoma (OTSCC) suggested that UTX expression in tumor tissues may predict poor survival outcomes in theses patients.^310^ In NSCLC cells, UTX is regarded as an oncogene which promotes cell proliferation and migration, and its expression is modulated by the EGFR-STAT3 axis.^311^ The promoting effect of UTX on lung cancer oncogenesis is mainly mediated through the upregulation of EZH2, and the UTX-deficient lung cancer is preferentially sensitive to EZH2 inhibitors.^312^ In addition, immunohistochemistry staining results revealed that UTX was highly expressed in CRC tissues and promotes CRC cell proliferation and maintains G0/G1 cell cycle progression via upregulating KIF 14 and pAKT.^313^ UTX positively regulates E-cadherin expression via modulating H3K27 demethylation and acetylation, activating the transcription of the E-cadherin at its promoter regions.^314^

In breast cancer, depletion of UTX resulted in upregulation of Myc-dependent expression of EMT factors, including SNAI and ZEB1/2.^315^ Thus, by forming a transcriptional repressive complex with LSD1, HDAC1 and DNMT1, UTX is a tumor suppressor and a negative regulator of EMT-induced CSC-like properties in breast cancer.^314^ However, pro-tumor functions of UTX were observed in the ER + subtype of breast cancer cells where the transactivation of UTX and estrogen receptor (ER) forms a feed-forward loop in response to hormone treatments.^316^

UTY on the other hand, is less frequently mutated in cancer than UTX^317^ and had less tumor-suppression effect than UTX.^309,318,319^ It was recently reported that UTY displayed weaker tumor-suppression abilities than UTX in leukemia, which was further reduced by the deleting the UTY cIDR (residues 498–795).^320^ Similar to UTX, the depletion of UTY promoted cell proliferation of urothelial bladder cancer cells^321^ and 12% of urothelial bladder carcinomas were identified with the absence of UTY.^322^ Moreover, the knockout of both UTX and UTY had a synergistic effect on the increase of proliferation, which might be attributed to the loss of dosage-dependent suppression effect of UTX/UTY in urothelial cancer.

## JMJD proteins in inflammation

**Fig. 4:**
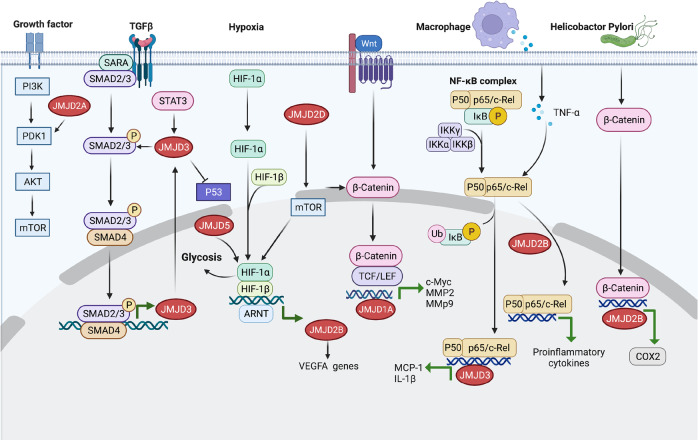
The signaling pathways involved in the regulation of cancer and inflammation by JMJD family members and their crosstalks. Figure was created with Biorender (www.bioender.com)

Histone modifications lead to significant alterations in genome structures and functions. Figure 4 presents the signaling pathways involved in the regulation of cancer and inflammation by JMJD family members and their crosstalks.

### JMJD1 and JMJD2

Under hypoxic conditions, the increased HIF-1α expression promotes the inflammatory injury of endothelial cells, which is independent on the NF-κB pathway. In JMJD1A-knockdown human umbilical vein endothelial cells (HUVECs), a number of genes involved in inflammation and the oxidative stress pathways were significantly downregulated.^323^

The involvement of JMJD2 in inflammation is best represented by JMJD2D which mediates inflammatory responses elicited by cytokines such as tumor necrosis factor α (TNFα), and consequently reshapes the immune microenvironment. TNFα is a pro-inflammatory cytokine produced by monocytes during acute inflammation and is implicated in a range of events leading to cell necrosis or apoptosis.^324^ TNFα was able to induce JMJD2D expression in dendritic cells and macrophages,^23^ and the demethylation of H3K9 by JMJD2D in turn participated in the TNFα response.^325^ In response to colon injury caused by inflammatory bowel disease (IBD), TNF-α secreted by macrophages activates the NF-κB signaling, upregulating JMJD2D in the colon epithelial cells.^326^

The expression of JMJD2B is significantly upregulated in gastric epithelial cells during *H. pylori* infection via β-catenin signaling.^327^ β-catenin directly binds to the promoter region of JMJD2B gene and activates its transcription. The upregulated JMJD2B, together with NF-κB, binds to COX-2 promoter to stimulate its transcription via demethylation of H3K9me3.^327^ Vascular inflammation is regarded as an preliminary step towards multiple human diseases, and its contributing factors remain incompletely defined. A recent study investigated the epigenetic changes during the transformation of vascular smooth muscle cells (VSMCs) into osteoblast-like cells in response to inflammation, and found that JMJD2B was the downstream target of IL-6/STAT3 axis, suggesting the pathogenic role of JMJD2B during chronic inflammation.^328^ In a LPS-induced vascular inflammation model, JMJD2A promotes the transactivation of pro-inflammatory cytokines, facilitating the binding of SET1A to NF-κB promoter.^329^

### JMJD3

As discussed earlier, JMJD3 together with UTX and UTY, belongs to the KDM6 family. However, as UTX and UTY are mainly involved in developmental processes, JMJD3 is implicated in regulating of inflammation and cellular senescence.^330–332^ JMJD3 is usually expressed at a low levels under normal conditions, but its expression is drastically increased by inflammatory stresses such as hypoxia inducers and oncogenic factors.^333–336^

JMJD3 interacts with distinct transcription factors and potently promotes the expression of inflammatory genes through H3K27me2,3 demethylation.^337,338^ One such example is the JMJD3-induced activation of NF-κB signaling genes.^339–341^ The knockdown of JMJD3 in human monocytic cells altered the expression profile of inflammatory genes in including chemokines, CD40 signaling and NF-κB-related inflammatory genes. In particular, JMJD3 knockdown and the subsequent enrichment of H3K27me3 at the promoter regions of NF-κB signaling genes suppresses the transcription of genes such as such as monocyte chemoattractant protein-1 (MCP-1) and IL-1β.^340^ In glomerular mesangial cells, the activation of the NF-κB/JMJD3 signaling pathway could promote high glucose-induced inflammation.^342^

JMJD3 also participates in the transcription process independent of its demethylase activity.^343,344^ For instance, STAT1 and STAT3 stimulated the transcription, which subsequently enhanced the expression of Lipopolysaccharide (LPS)-induced inflammatory genes.^345^ Besides, JMJD3 is also implicated in the SMAD3-mediated TGF-β signaling pathway.^346^

JMJD3 is involved in several inflammation-related diseases such as rheumatoid arthritis (RA)^347,348^ where JMJD3 modulates the inflammation persistence and angiogenesis of RA via transcription factor GATA4.^349^ Given that the H3K27 level is elevated in the midbrain of aged mice, JMJD3 might reshape the immune microenvironment of Parkinson’s disease.^350^ In Parkinson’s disease, JMJD3 enhances the M1 pro-inflammatory response by suppressing the anti-inflammatory microglia M2 phenotype, resulting in increased neuronal cell death.^350,351^ In diabetic peripheral tissues, JMJD3 mediates the chronic activation of macrophages, providing another rationale for using histone demethylase inhibitors for the treatment of nonhealing diabetic wounds.^352^

JMJD3 also plays a pivotal role in cell response to bacteria, parasites, or virus infection. JMJD3 modulates the recovery of murine macrophages from exposure to the lethal anthrax toxin.^353^ During the latency stage of herpes simplex virus 1 (HSV-1) infection, JMJD3 prevents the reactivation of HSV-1 in sensory neurons by decreasing H3K27me3.^354^ JMJD3 deficiency in CD4+ T cells leads to the accumulation of T cells in the thymus, and reduced T-cell trafficking to the secondary lymphoid organs. The underlying mechanism for the regulation is the binding of JMJD3 to the Pdlim4 promoter which modulates its expression to affect T cell trafficking.^355^

### JMJD6

JMJD6 is most highly expressed in innate immune cells. One of its target genes by its arginine demethylase activity is tumor necrosis factor receptor-associated factor 6 (TRAF6), which can be both methylated and demethylated at different arginine sites.^181^ TRAF6 could be involved in the pathogenesis of a variety of autoimmune diseases,^356^ and the reversible arginine methylation status of TRAF6 by JMJD6 thus provides a novel mechanism for regulation of innate immune pathways. A recent study reported a potential underlying mechanism for the pathogenesis of neuropathic pain. The overexpression of JMJD6 suppressed the activation of NF-κB signaling peripheral nerve injury, suggesting its therapeutic value in neuropathic pain.^357^ JMJD6 is also involved in viral RNA replication. Immunoprecipitation assays confirmed a physical interaction between recombinant JMJD6 and DHX9,^179^ which is required to replicate the foot-and-mouth disease virus (FMDV) in cells.^358^

The impact of JMJD6 on transcriptional regulator Aire reflects its critical role in the spontaneous development of multi-organ autoimmunity in mice, such as thymus and T cell development.^359^ For example, in patients with chronic hepatitis B virus infection, T lymphocytes usually experience a decrease in JMJD6 expression.^360^ It is thus postulated that the “exhausted” T cells when exposed to chronic inflammation can be partially attributed to aberrant JMJD6 expression. In fact, the deficiency of JMJD6 in normal peripheral blood mononuclear cells specifically inhibited CD4+ T cell proliferation and is associated with an increased level of cyclin-dependent kinase inhibitor 3 (CDKN3),^360^ a suppressor of cell cycle progression.^361^ However, it remains incompletely defined whether the activity of JMJD6 in T cell exhaustion is cell autonomous.^362^

### JMJD8

A recent gene expression profiling analysis demonstrated the highly enriched expression of JMJD8 in adipocytes, which is affected by metabolic and nutritional status.^363^ JMJD8 expression in turn exerts its regulatory effect on the expression of a series of pro-inflammatory genes, thereby triggering inflammation responses. Importantly, functional interaction between JMJD8 and IRF3, a pro-inflammatory factor involved in adipocyte inflammation and insulin sensitivity, suggested that JMJD8 might be a junction bridging adipocyte insulin sensitivity and inflammation.^363^ Moreover, JMJD8 functions as a positive regulator of TNF-induced NF-κB signaling,^205^ which regulates a large array of genes involved in multiple immune and inflammatory responses.^364^

### MDIG

Despite conflicting results regarding the impact of MDIG on Th2 development, it has been well established that MDIG is involved in Th2 response-related atopic asthma and parasitic helminth infection. A genetic case-control study suggested that the T allele of MDIG correlated with an increased risk of atopic asthma, a disease typically driven by pulmonary inflammation.^365,366^ MDIG deficiency extenuates airway hyper-responsiveness and pulmonary inflammation, possibly by controlling IL-4 production.^367^ MDIG may also promote silica-induced lung fibrosis by altering the balance between Th17 and Treg cells.^368^ More recently, MDIG mediates the response to environmental exposure to COVID-19, making it a therapeutic target of COVID-19 ameliorates the pulmonary symptoms.^369^ Further studies are warranted to elucidate the underlying mechanisms for the involvement of MDIG in pulmonary inflammation.

### JARID2

The role of JARID2 in inflammation is best characterized by its function in Crohn’s disease (CD), the most common type of inflammatory bowel disease. An intricate series of pathological factors are associated with the onset of CD, but the exact molecular mechanisms remain incompletely defined.^370,371^ Regulatory B cells producing IL-10 facilitate intestinal homeostasis, which potently inhibits mucosal inflammatory responses of intestines.^372^ Patients with Crohn’s disease have a decreased level of regulatory B cells^373,374^ and the deficiency in B10 cells is reportedly related to CD development.^375^ A recent study demonstrated the increased IL-10 production by B cells mediated by JARID2 which promotes H3K27me3 binding to the IL10 promoter regions. This provides a novel molecular explanation for the pathogenesis of B10 cells in CD patients.^376^

Another study introduced a novel mechanism through which inflammatory cytokine interferon-γ (IFN-γ) and class II transactivator (CIITA) collectively reset the fate of post-inflammation muscle cells.^377^ IFN-γ has been found to prevent muscle development during inflammation.^378^ Circulating IFN-γ increased PCR recruitment in a JARID2-dependent manner, thereby suppressing muscle-specific genes. Moreover, as one of the target genes of miR-155, JARID could attenuate theTh2 and Th17-mediated airway inflammation.^379^

### UTX

As described earlier, it remains to be elucidated whether UTX expression contributes to the female predominance of autoimmune diseases. Previous analyses investigated UTX expression in CD4+ T lymphocytes of female versus male mice,^380,381^ and suggested that the X escape of UTX is involved in a wide range of immune response genes, providing a potential explanation for the female susceptibility to autoimmune disease.^382^ The role of UTX in innate immune responses described so far relies on its H3K27me2/3 demethylation activities in macrophages, which promotes pro-inflammatory cytokine transcription such as IL-6 and IFN-β.^383^ NF-κB signaling can be activated by UTX, leading to increased secretion of macrophage migration inhibitory factor in neural stem cells.^384^ Thus, UTX could be recognized as a protecting marker that improves neurological function recovery after spinal cord injury.

Besides, UTX is necessary for the differentiation of CD4+ T cells to Tfh cells during chronic virus infection. The depletion of UTX in mice promoted H3K27 methylation level, decreased the gene expression at Tfh-related genetic loci, and led to deficient virus-specific IgG production.^385^ UTX gene mutations are often associated with bladder cancer. Recently, the absence of UTX was reported to induce activation of inflammatory pathways that contributes to bladder cancer in cooperation with p53 dysfunction.^386^ The loss of UTX in CD4+ T cells also aggravates allergic contact dermatitis in mice.^387^

## JMJD proteins as therapeutic targets

Given that the JMJD class of histone demethylase is involved in various physiological and pathological processes, specific JMJD inhibitor would be an attractive strategy, the utility of which should not be limited to combating cancer but also the treatment of inflammatory disorders such as asthma. However, due to its highly polar 2-OG binding pocket, the development of small-molecule inhibitors for the JMJD family has lagged behind, with several JMJD inhibitors being reported but functionally inactive.^388,389^ We summarized known inhibitors targeting JMJD family proteins evaluated for the treatment of cancer and inflammatory disease (Table 2).

**Table 2:** Inhibitors targeting JMJD family proteins evaluated for the treatment of cancer and inflammatory disease

Inhibitor	Function in cancer	Function in inflammation
**JMJD2**		
NCDM-32B	Inhibits cell growth of basal breast cancer cell lines	
QC6352	Inhibits proliferation and overcomes therapeutic resistance of breast cancer cells	
TACH101	A pan inhibitor of JMJD2 subfamily	
JIB04	Overcomes cisplatin resistance of lung cancer	
ML324	Overcomes cisplatin resistance of lung cancer	Anti-viral activity against both herpes simplex virus (HSV) and human cytomegalovirus (hCMV) infection
Caffeic acid	Under clinical trial for the treatment of esophageal cancer (NCT03070262)	
**JMJD3/UTX**		
GSK-J4	Antitumor efficacy in glioma, leukemia, breast, prostate, lung cancer, hemangiosarcoma, Ewing sarcoma and chondrosarcoma	
	Combination partner for deacetylase inhibitor panobinostat in glioma and decitabine in leukemia	
	Radiosensitization of diffuse intrinsic pontine glioma	
**JMJD6**		
WL12	Inhibits JMJD6 enzymatic activity and JMJD6-dependent cell proliferation	
SKLB325	Suppresses the proliferation and induces cell apoptosis of ovarian cancer	
	Sensitizes renal cell carcinoma cells to sunitinib and work synergistically with sunitinib	
J2	Highly selective JMJD6 inhibitor with minimal activity against other JMJD family proteins	
**JARID1**		
1,7-naphthyridones	Selective to JARID1 over JMJD2 related isoforms	Achieves the same efficacy with dexmedetomidine on acute kidney injury
CPI-455	JARID1-specific inhibitor that reduces the stem cell-like features of oral squamous cell carcinoma cells	
	Reduces drug tolerant persister (DTP) cells in cancer models	
	Effective in temozolomide (TMZ)-resistant glioblastoma	
KDOAM-25	Inhibits proliferation of multiple myeloma cells	
Ryuvidine	Exerts inhibitory activity on JARID1A but also recombinant JARID1B and C	
KDM5-inh1	A novel panel of selective JARID1 inhibitors that is especially effective in HER2 + breast cancer	

### JMJD2 inhibitor

An early study described a variety of inhibitor scaffolds with the capacity to suppress 2-OG-dependent JMJD2 histone demethylases, which would facilitate the establishment of small-molecule probes for the identification of enzyme functions in epigenetic signaling.^390^ Known JMJD2 inhibitors can be classified as either 2-OG cofactor mimics, substrate-competitors, metal cofactor inhibitors, and peptide inhibitors.^391^ Cofactor mimics competitively binding to Fe(II) at the catalytic site of JMJD2 proteins and modifies the availability of the 2-OG cofactor required for cancer cell metabolism. This class of JMJD2 inhibitors includes fumarate and succinate, which have long been identified as 2-OG antagonists.^392^

As the overexpression of JMJD2 is frequently observed in breast cancers, previous studies mainly analyzed the therapeutic potential of JMJD2 inhibitors in breast cancer. For instance, a JMJD2 inhibitor, NCDM-32B, effectively decreased cell growth of basal breast cancer cell lines.^66^ With structure-based drug design, a novel JMJD2 inhibitor QC6352 was developed, which potently suppressed the proliferation, sphere formation, and in vivo tumor growth of TNBC, as well as PDX models of colon cancer.^389^ Moreover, QC6352 abrogated EGFR expression, thereby overcoming therapeutic resistance in breast cancer.^393^ Recently TACH101, a pan inhibitor of the JMJD2 subfamily was introduced. This compound exhibited high inhibitory efficacy on four KDM4 isoforms (A-D) and was able to induce cell apoptosis of esophageal cancer, TNBC, and CRC cell lines. Animal studies presented a 4.4-fold lower tumor-initiating cell frequency by TACH101.^394^ In lung cancer, JMJD2 inhibition by either JMJD2 selective inhibitor ML324 or pan-JMJD inhibitor JIB04 could overcome cisplatin resistance, potentially by preventing ATR-Chk1 replication checkpoint.^395^ Furthermore, the combined treatment of JMJD2 inhibitors and LSD1 inhibitors may represent a more effective strategy for the enhancement of chemotherapy efficacy.^396^

The 5-chloro-8-hydroxyquinoline (5-c-8HQ), also referenced under CAS 5852-78-8, is a well-studied JMJD2D inhibitor used in multiple researches. The treatment of 5-c-8HQ in mice leads to significantly smaller and fewer colitis-associated tumors.^94^ JMJD2D inhibition using 5-c-8HQ decreased the self-renewal capacities of liver cancer stem-like cells, thereby suppressing live cancer progression.^397^ It has also been reported that 5-c-8HQ works in synergy with Hedgehog inhibitor vismodegib to suppress CRC tumorigenesis and cell proliferation.^326^

A group of tumor-initiating cells (TIC) were isolated from patient samples of esophageal squamous cell carcinoma (ESCC) which is characterized by stem cell-like features. Importantly, JMJD2C expression was upregulated in this subpopulation, suggesting the potential of JMJD2C inhibition in eliminating ESCC TIC compartment.^398^ JMJD2C was reported to confer stem-cell-like characteristics in ESCC cells and caffeic acid (3,4-dihydroxycinnamic acid, CA) is able to suppress its demethylation activity.^399^ An ongoing clinical trial aims to investigate the efficacy and safety of caffeic acid for the treatment of esophageal cancer (NCT03070262).^400^ It is the only clinical trial up to date that is registered on www.clinicaltrials.gov to assess the efficacy of JMJD protein inhibitors for cancer treatment. In this trial, 240 patients with advanced esophageal squamous cell cancer (ESCC) were randomized into two arms: coffeic acid treatment (300 mg, tid, po) or placebo treatment. Patients will be followed every year and the clinical outcomes will be recorded as overall survival and progression-free survival. The application of JMD2 inhibitor is not limited to cancer treatment. ML324 has potent anti-viral activity against both herpes simplex virus (HSV) and human cytomegalovirus (hCMV) infection and recurrence, suggesting the therapeutic value of chromatin-based inhibitors against viral infection.^401,402^ ML324 was also tested for the treatment of the depression-like condition in mice by increasing the repressive histone methylation in the nucleus accumbens.^403^

### JMJD3 and UTX inhibitor

A research team reported the first selective JMJD3 inhibitor, supporting its role as a therapeutic target in epigenetic drug discovery. With the optimization of a series of compounds obtained from the screening of a compound collection,^404^ a JMJD3 and UTX specific inhibitor GSK-J1 was developed with a half-maximum inhibitory concentration (IC50) of 60 nM.^405,406^ GSK-J3 was refined based on GSK-J1 with substitution at the para position to the pyridine nitrogen and improved access to solvent. Later, the acid groups of GSK-J1 and GSK-J2 were concealed with ethyl esters, which derived new compounds GSK-J4 and GSK-J5.

The inhibition of JMJD3 by GSK-J4 increased the level of H3K27 methylation and demonstrated potent antitumor efficacy in glioma and leukemia where the H3K27me dysregulation occurs recurrently.^122,407,408^ The underlying mechanism for the potent efficacy of GSK-J4 in leukemia might be its downregulation of Cyclic-AMP response element-binding protein.^409^ Recent evidence suggested a potential combinatory effect of GSK-J4 and decitabine in leukemia cells by inducing cell cycle arrest, cell apoptosis and PKC-α/p-bcl2 pathway inhibition.^410^ In glioma cells, GSK-J4 is a combination partner for deacetylase inhibitor panobinostat in glioma cells, and for doxorubicin in KRAS-mutant anaplastic thyroid cancer.^411,412^ GSK-J4 is also involved in the radiosensitization of diffuse intrinsic pontine glioma, an aggressive pediatric brainstem tumor by inducing DNA repair deficiency.^408^ Other cancers that responded to GSK-J4 included BC,^413^ PC,^131^ lung cancer,^414^ hemangiosarcoma,^415^ SMARCA4-mutant cancer,^416^ Ewing sarcoma,^417^ and chondrosarcoma^418^ where GSK-J4 displayed potent antitumor effect.

GSK-J4 cannot be perceived solely as an antitumor drug, as it is also applied for the treatment of inflammatory diseases such as inflammatory colitis by suppressing the inflammatory potential and increasing the generation of tolerogenic dendritic cells.^419^ GSK-J4 selectively reduced intracellular labile iron in dopaminergic neurons, and this neuroprotection is based on its epigenetic mechanism, suggesting the therapeutic potential of GSK-J4 for Parkinson’s disease.^420^

### JMJD6 inhibitor

Given that JMJD6 has been reported with both demethylation and hydroxylation activities, both of which require the presence of Fe (II) and 2-OG and occur at the same active sites, it is thus speculated that the inhibition of JMJD6 can rely on the targeting of either arginine demethylation or lysyl hydroxylation.

To date, three JMJD6 inhibitor candidates have been proposed which all remain at the preclinical stage. The first JMJD6-targeting inhibitor, WL12, was developed following silico protocol by targeting the druggable 2OG-binding site. The inhibition of JMJD6 enzymatic activity by WL12 lead to decreased cell proliferation.^421^ Another JMJD6 inhibitor, SKLB325, significantly suppressed the proliferation and induced cell apoptosis of ovarian cancer in a dose-dependent manner. The study further suggested the colocalization of JMJD6 with p53 in the nucleus, upregulating p53 and its downstream effectors.^204^ SKLB325 also sensitizes renal cell carcinoma cells to sunitinib and works synergistically with sunitinib in inhibiting RCC growth.^422^ Recently, a research team performed molecular docking and retrieved a new JMJD6 inhibiting compound J2, the optimization of which yielded a more potent JMJD6 inhibitor 7p. The IC50 value of 7p against JMJD6 was 0.681 μM, with minimal activity against other JMJD family proteins.^423^

### JARID1 inhibitor

The contributions of JARID1 to cancer progression have derived respective countermeasures targeting JARID1. A series of pan-JARID1 inhibitors were optimized from compound 1, a hit initially designed to specifically target JARID1C. The optimization of compound 1 led to compound 20 which is highly selective for JARID1 enzymes and able to induce a global increase in H3K4me3 level.^424^ Another study combined a high throughput screening hit with an established scaffold, and developed a novel JARID1 inhibitor 1,7-naphthyridones, which is more selective to JARID1 over JMJD2 related isoforms.^425^

The first JARID1-specific inhibitor is CPI-455, with 200-fold higher selectivity for JARID1 than for JMJD2.^426^ The inhibition of JARID1B by CPI-455 reduced the stem cell-like features of oral squamous cell carcinoma cells, but cells also displayed demethylase-independent activities refractory to inhibition.^266^ JARID1A is highly expressed in drug tolerant persister (DTP) cells, a subpopulation of tumor cells that contributes to the growing number of drug resistant cells^427^ such as TMZ-resistant glioblastoma cells.^428^ Given that drug tolerance of tumor cells was partially dependent on demethylase activity, CPI-455 was used to reduce DTPs in multiple models.^426^ CPI-455 is more effective in temozolomide (TMZ)-resistant glioblastoma cells than in TMZ-native cells. Thus, CPI-455 may be a sensitizing agent for TMZ in glioblastoma, indicating the combinational potential of targeting the epigenetic landscape with cytotoxic therapies.^429^ Likewise in leukemia, CPI-455 treatment sensitized acute promyelocytic leukemia (APL) cells to all-trans retinoic acid-induced differentiation.^430^ Importantly, CPI-455 may also be applied in the context of inflammatory diseases. Dexmedetomidine (DEX) is frequently used to prevent excessive inflammatory response in sepsis-induced organ failure. In a mouse model with acute kidney injury, DEX and CPI-455 achieved the same effect in decreasing H3K4me3 enrichment of multiple inflammatory cytokine genes. Thus, DEX can be used to attenuate acute kidney injury by blocking JARID1 during sepsis.^431^

Another JARID1 inhibitor KDOAM-25, has a half maximal inhibitory concentration of <100 nM for JARID1A-D, and demonstrates no off-target effect on a panel of 55 other enzymes. As discussed earlier, JARID1B is an oncogenic factor for multiple myeloma. The treatment of multiple myeloma cells with KDOAM-25M led to decreased cell proliferation and increased global H3K4 methylation level at transcription sites.^432^ Furthermore, ryuvidine might be a lead compound for JARID1-targeting therapeutics, which exerted its inhibitory activity on not only JARID1A but also recombinant JARID1B and C.^433^ Recent research assessed the antitumor effect of KDM5-inh1, a novel panel of selective JARID1 inhibitors in multiple cancer cell lines, and found that JARID1 inhibition is especially effective in HER2 + breast cancer, which might serve as a diagnostic tool for the selection of target patients.^434^

## Conclusion and future perspectives

JMJD protein family members regulate multiple tumor-associated genes either dependent or independent of its histone demethylase activity according to different cellular contexts. Growing evidence has suggested the diverse functions of JMJD class of histone demethylase in pathological processes, justifying the development of small-molecule inhibitors against JMJD proteins. The utility of JMJD protein inhibitors should not be limited to combating cancer but also the treatment of inflammatory disorders such as asthma. However, several obstacles need to be overcome in the application of JMJD proteins as treatment targets for cancer and inflammatory diseases.

First, as some of them promote cancer progression, their overexpression or the activation of their enzymatic functions could be hallmarks of tumorigenesis. Nevertheless, the cancer-suppressive activities have also been implicated for some JMJD family members such as JMJD3 which exhibits a dual role in CRC progression, making it both a tumor suppressor and a tumor activator. Thus, before using JMJD protein blockade for treatment, it is important to elucidate the specific functions of each JMJD protein in cancer under different conditions.

Secondly, due to its highly polar 2-OG binding pocket, the development of small-molecule inhibitors for the JMJD family has lagged behind, with several JMJD inhibitors being reported but functionally inactive. Moreover, despite growing research, no known inhibitors to date are commercially available for the treatment of any cancer type. For instance, various JMJD2 inhibitors have been reported as cancer therapeutic agents, but currently there is only one agent under clinical evaluation. To eradicate non-selective target effects and improve the selectivity of JMJD inhibitors, further studies on the structural information and structure-activity relationship of JMJD proteins are warranted.
